# Decalogue for mastering robotic transanal minimally invasive surgery (rTAMIS)

**DOI:** 10.1007/s10151-024-02957-9

**Published:** 2024-07-16

**Authors:** H. Guadalajara, M. Leon-Arellano, J. L. Dominguez-Tristancho, D. García-Olmo

**Affiliations:** 1 Autonomous University of Madrid, Madrid, Spain; 2 Department of General and Digestive Surgery, University Hospital Fundación Jiménez Díaz, Avenida Reyes Católicos, 2, 28040 Madrid, Spain

**Keywords:** Transanal robotic surgery, Rectal cancer, rTAMIS, Local excision, Minimally invasive surgery

## Abstract

This manuscript offers a detailed description of our successful tips for mastering transanal robotic surgery. It covers various aspects, including patient positioning, management of abdominal pressures to maintain a stable pneumorectum, platform positioning, camera alignment, trocar positioning to minimize collisions, instruments used, and approaches to tumor resection.

## Introduction

Since local excision began, the technique has evolved significantly, and so have its indications. In 1982, Dr. Klaus Buess introduced transanal endoscopic microsurgery (TEM), a minimally invasive surgical technique, through a groundbreaking paper and patented device known as TEM, which utilizes a rigid rectoscope [1]. This device played a pivotal role in the widespread adoption and refinement of the technique. However, its complexity eventually led to its gradual replacement by simpler devices. The next major innovation was the TEO, developed by Karl Storz, which also relied on a rigid endoscope. Now, the GelPOINT Path® appears to be the future of transanal surgery (TAMIS). Unlike its predecessors, it is not a rigid endoscope but rather a single-port device, making it much easier to use. In summary, TEM, TEO, and TAMIS represent the most significant techniques and devices developed for transanal surgery. TAMIS currently holds the dominant position in this field.

Advancements in robotic technology continue to evolve, with the imminent arrival of single-port devices like the da Vinci SP® system poised to revolutionize transanal surgery [2]. However, as we eagerly anticipate these developments, we currently rely on adapting existing platforms such as the Xi system to navigate the constraints of narrow anatomical spaces for which they were not originally designed. Considering this necessity, this publication aims to share invaluable insights that have greatly enhanced our proficiency in mastering this approach.

## Transanal robotic surgery applications

Robotic transanal minimally invasive surgery, also called rTAMIS, is used to treat various conditions affecting the lower rectum and anal canal. Below is an overview of its indications.

### Early-stage rectal cancer

rTAMIS is a technique used to remove early-stage rectal tumors or polyps, especially those in the mid to lower rectum. Studies have shown that it can achieve comparable oncological outcomes to traditional open or laparoscopic approaches while offering the benefits of minimally invasive surgery [3, 4].

### Repair of dehiscent low rectal anastomosis

Extrapolating from the experience with TAMIS, we consider that rTAMIS is an effective technique for repairing dehiscent colorectal anastomoses in the early stages [5].

### Advanced rectal cancer

While some surgeons adhere to the conventional approach to rectal cancer surgery supplemented by robotics, the field has undergone a revolution that challenges this traditional paradigm. Organ preservation strategies are successful in more than 50% of cases, and radical surgery is no longer the best option for everyone. This is where surgeons can still make a difference in rectal cancer treatment with local excision. The hot spot for surgeons is now in local excision [6].

Several studies have evaluated this choice: A study conducted by the Rome group [7], which randomized cT2N0 lower rectal tumors to radical surgery (total mesorectal excision, TME) or neoadjuvant chemoradiotherapy (NCRT) + local resection, found that after a 9-year follow-up, local recurrence rates were similar in both groups (6% vs 8%). These good results have been reproduced by other studies, such as the Dutch CARTS study [8], which achieved a 74% organ preservation rate. However, it is also mentioned that the effect of local resection after radiotherapy includes wound-related complications as well as up to 43% grade 3 chemoradiotherapy toxicity. Three other important studies have set the trend in this landscape, the French GRECCAR 2 [9], the American ACOSOG [10], and the Spanish TAU-TEM [11]. Finally, English groups published the TREC study results in 2021 and achieved an 88% margin-free rate and low toxicity (15% grade 3) in < 3 cm, cT1-2 lower rectal tumors that had been treated with short-cycle RT and local resection 8–10 weeks later [12].

### Other proctological diseases

rTAMIS may be useful for treating challenging rectovaginal fistulas [13] as well as neuroendocrine tumors [14].

## Tips

Robotic surgery is revolutionizing the surgical landscape, yet mastering the technique of local excision remains challenging. Surgeons can benefit from lesser-known tips and strategies when implementing this approach.

### Tip 1: position

Traditionally, the patient’s positioning was determined by the tumor’s location, ensuring the lesion remained in the lower portion of the surgical field. This practice was imperative when using rigid rectoscopes and proved beneficial with conventional TAMIS. The reasoning for this approach stemmed from the difficulties encountered in managing lesions positioned on the upper aspect of the surgical field. Operating on lesions located at the top of the surgical field is not only uncomfortable but also makes suturing the rectal defect afterward challenging.

However, with the advent of robotic approaches, surgical procedures have become significantly more versatile. Robotic systems offer enhanced maneuverability, enabling surgeons to achieve successful outcomes with greater ease. This technological advancement has particularly revolutionized the suturing and closure of wounds, overcoming the previous limitations.

Hence, the systematic jackknife position, set at 145° with open legs and a slight head-down inclination, stands out as our preferred orientation. This choice is not solely based on its feasibility but is also driven by its efficacy in sustaining a more stable pneumorectum. The abdominal compression achieved in this position serves to prevent the unintended diffusion of gas throughout the bowel.

As important as the jackknife position is, maintaining relaxation is also necessary to avoid straining that collapses the rectal space.

**Fig. 1:**
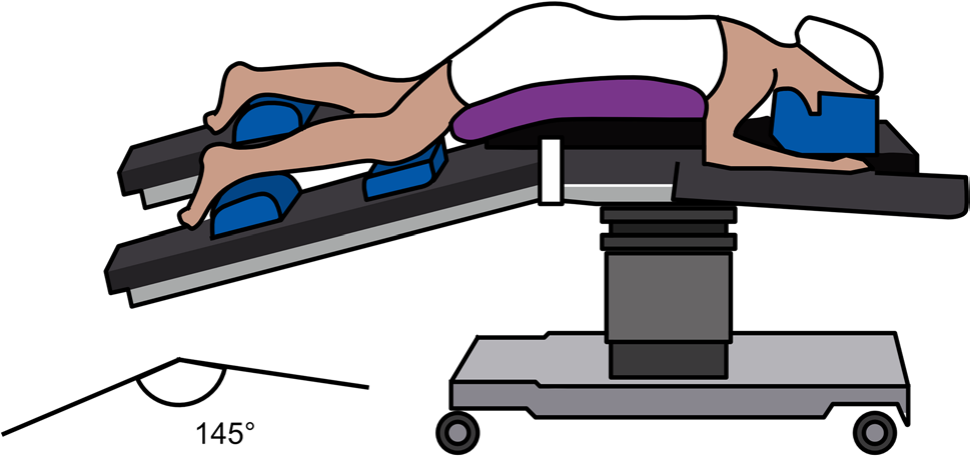
Patient in jackknife position

In patients undergoing femorofemoral bypass surgery, vigilance is crucial to address potential issues related to pubic compression, ensuring optimal leg perfusion following positioning (Fig. 1).

### Tip 2

**Fig. 2:**
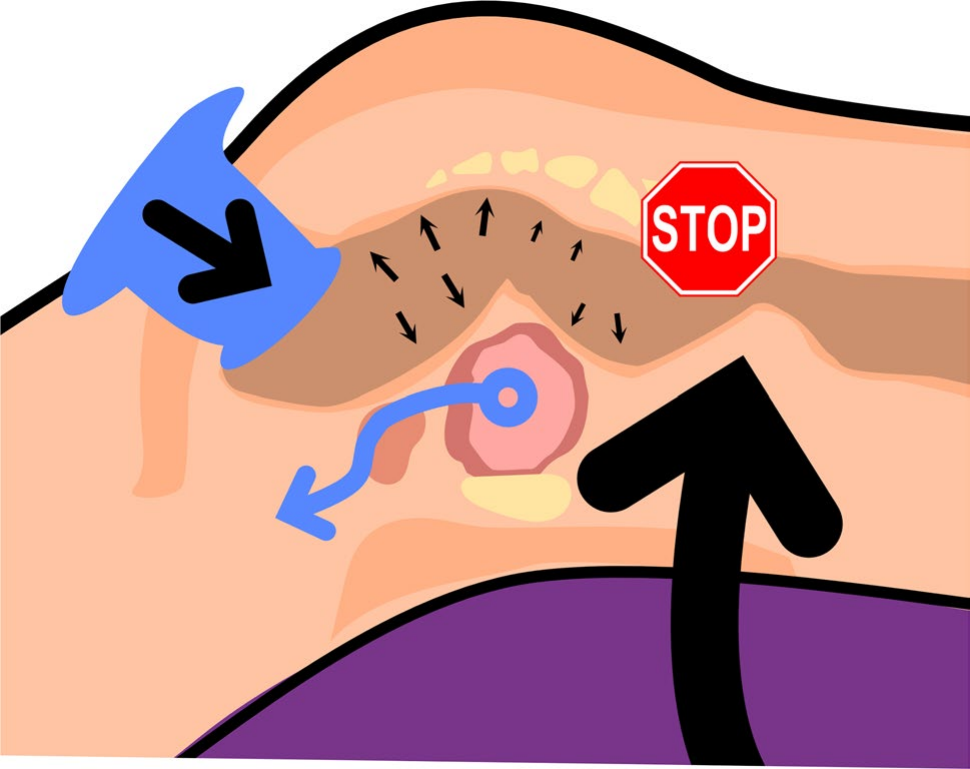
Pneumorectum pressure dynamics

Understanding the dynamics of intra-abdominal pressure is crucial in creating an optimal surgical field, as depicted in Fig. 2.

Although not strictly necessary from an anesthetic standpoint, the insertion of a bladder catheter can be beneficial by enlarging the pelvic space and facilitating rectal distension.

### Tip 3

**Fig. 3:**
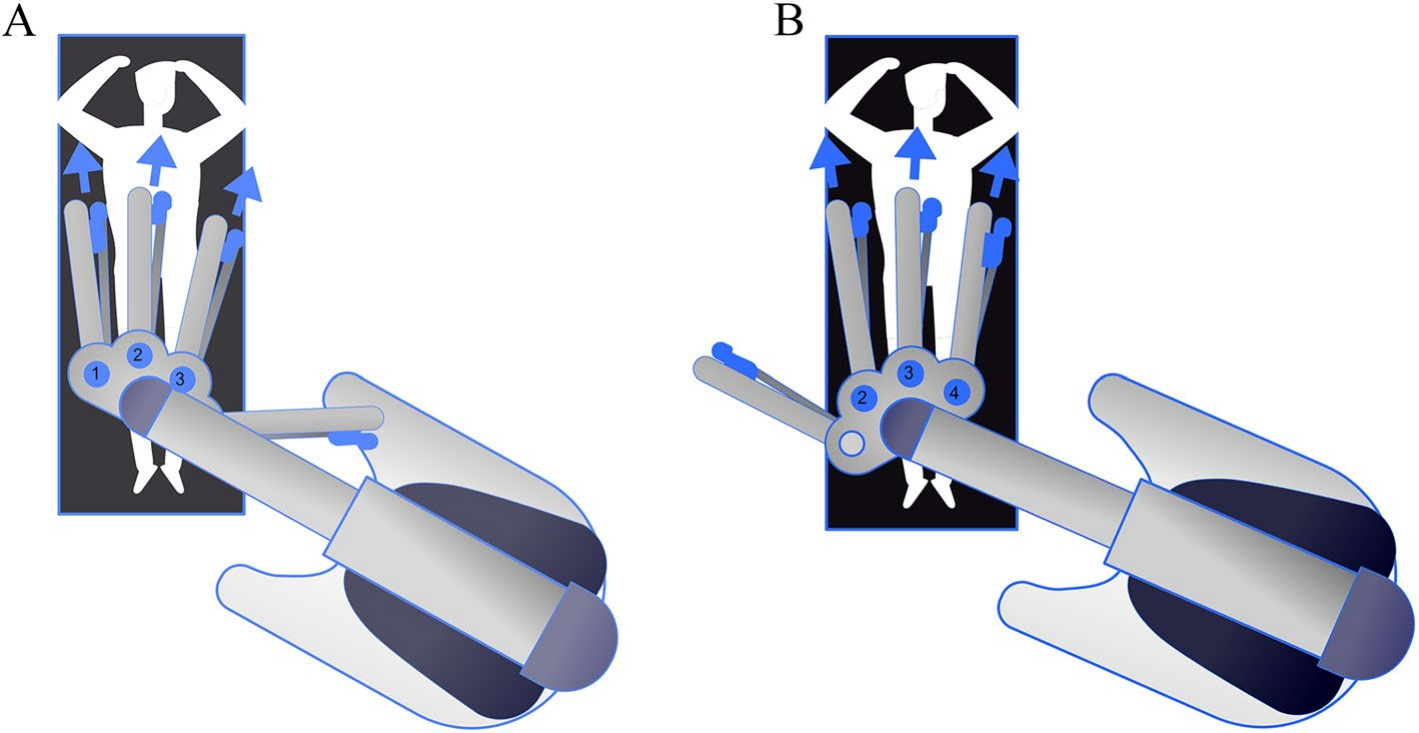
da Vinci Xi® docking in **a** 123 configuration and **b** 234 configuration

Let us delve deeper into the reasoning behind the choice of using arm 123 when da Vinci Xi® arms enter from the right side of the patient in upper abdomen configuration. See Fig. 3.

*Space considerations*

- Assistant accessibility: Positioning arm 123 creates more space on the left side of the patient, facilitating better accessibility for the surgical assistant. Additionally, small adjustments with the clearance button will reduce collisions. It also facilitates good access to the airway for the anesthetic team.
- Optimized platform stability: The utilization of arm 123 significantly enhances stability within the robotic platform throughout the entirety of the surgical procedure, eliminating the need for extreme arm rotations.

In summary, choosing arm 123 when the da Vinci Xi® arms enter from the right side in upper abdomen configuration is a strategic decision based on considerations of space, cart stability, and procedural efficiency. It optimizes the working environment for the surgical and anaesthetic team, and simplifies the overall robot-assisted surgical process (Fig. 3a). Figure 3b shows the 234 configuration.

### Tip 4

As a result of inherent limitations of the reduced space, automatic targeting is not feasible. As previously emphasized, a profound comprehension of arm positioning becomes paramount. Consequently, a manual targeting process is unavoidable, underscoring the importance of meticulous optical alignment with the patient’s spine.

### Tip 5

**Fig. 4:**
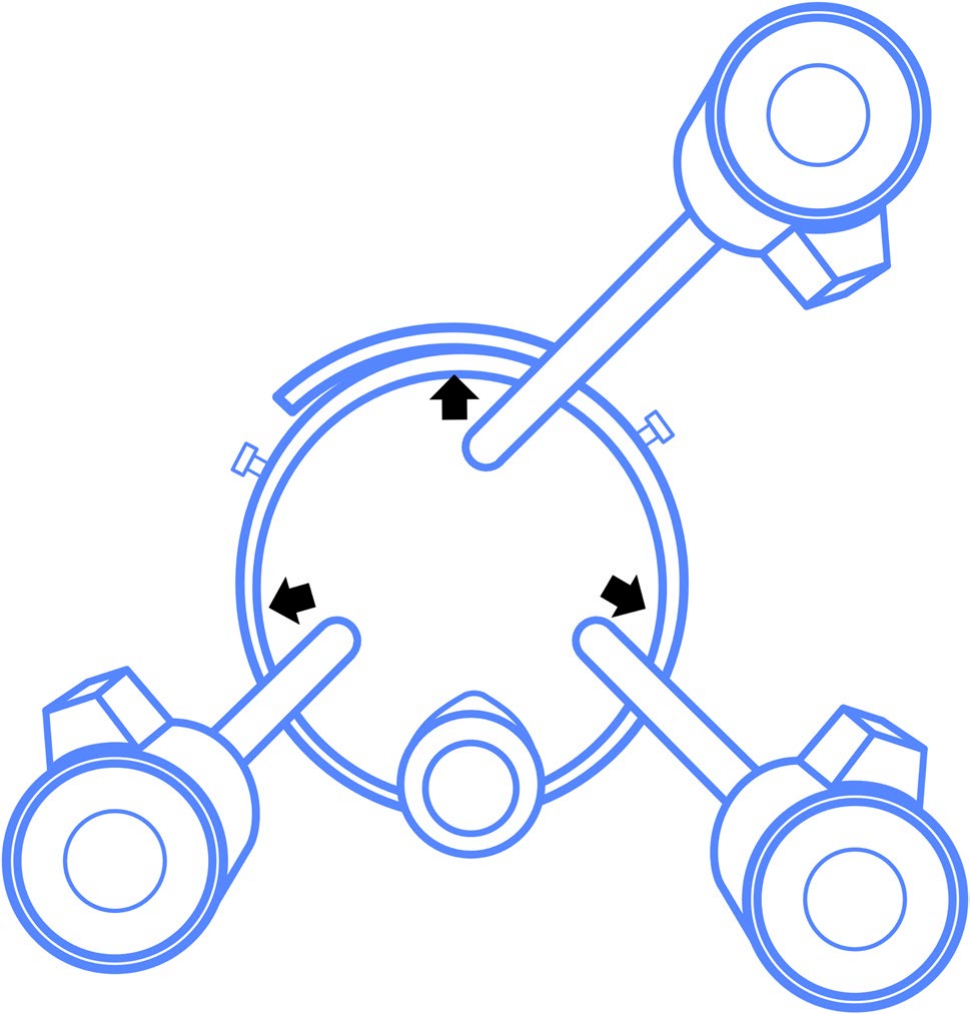
GelPOINT Path® trocar positioning. Frontal view

The GelPOINT Path® is best utilized with a triangular distribution of the trocars. At the apex, position the optic trocar, with the left and right working devices flanking it. The assistant port, dedicated to sutures and suction, is situated below. After completing the docking process, gently adjust the trocar positions outward towards the outer ring (Fig. 4).

### Tip 6

**Fig. 5:**
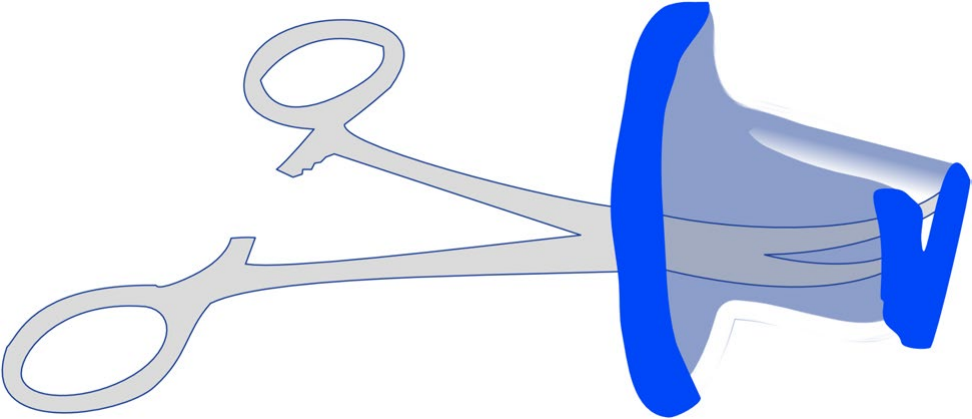
GelPOINT Path® insertion folding

For the GelPoint Path® insertion, the anal area must be highly lubricated and softly dilated before the insertion. The port needs to be folded as shown in Fig. 5 and gently inserted. Finally, once inserted, it needs to be unfolded.

### Tip 7

The implementation of a staggered docking technique proves advantageous in reducing collisions between ports. Trocar placement is performed before the cap is connected to the port. Then, after docking, the stepped configuration is achieved by pulling and pushing the trocars.

**Fig. 6:**
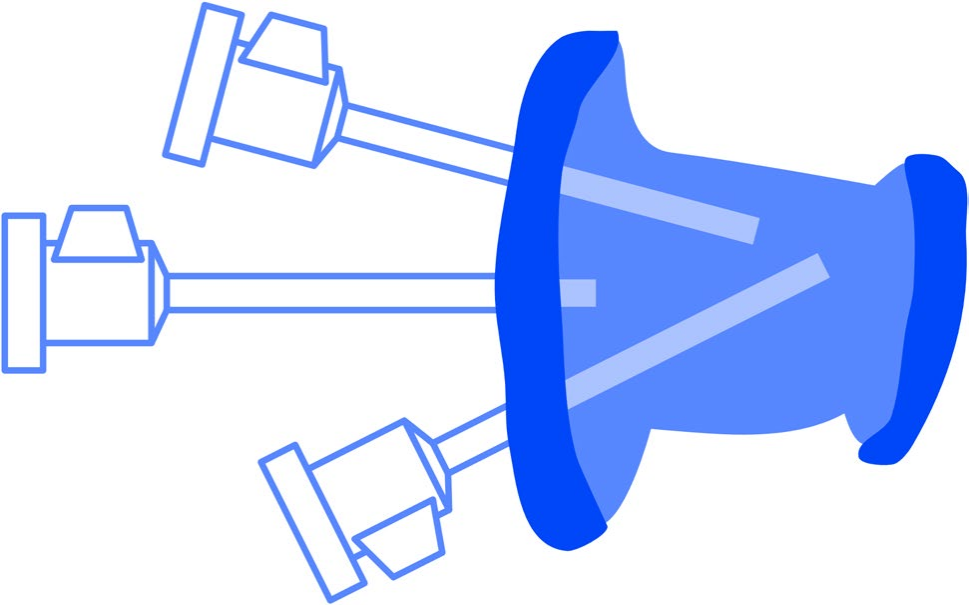
GelPOINT Path® trocar positioning. Lateral view

Generally, the optic port is positioned as the more exteriorized port in this setup in the upper position for posterior tumors and lower position for anterior ones (Fig. 6).

### Tip 8

**Fig. 7:**
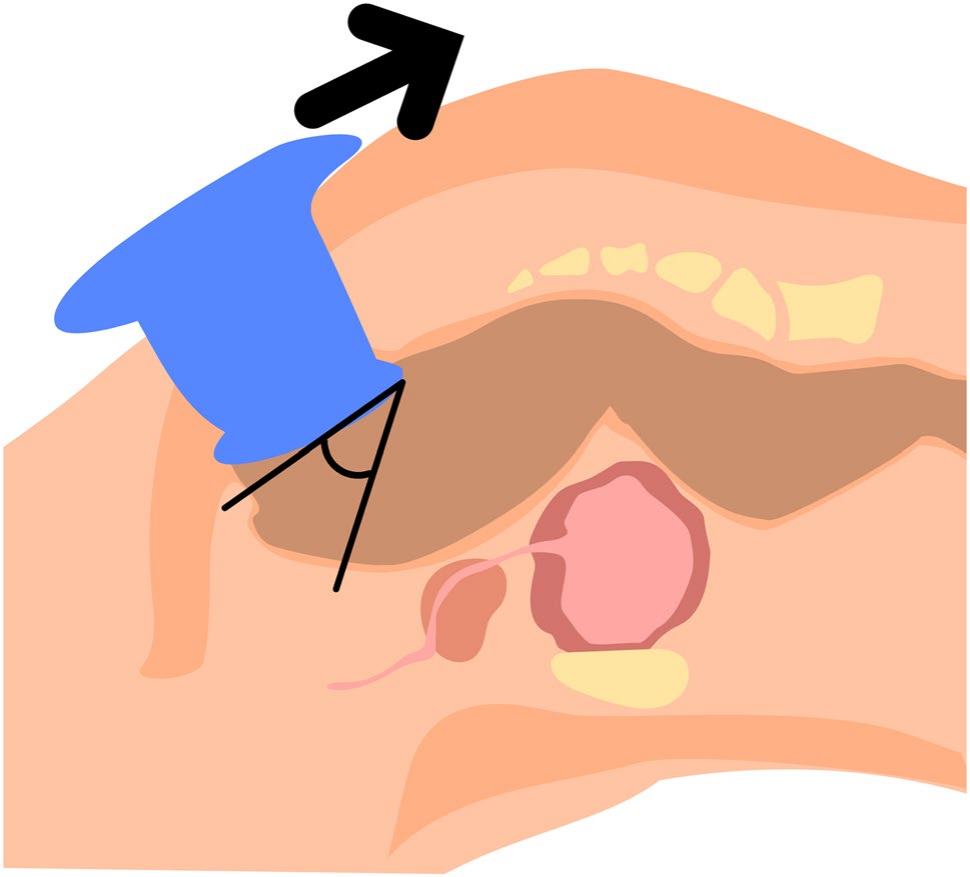
GelPOINT Path® movements

The GelPOINT Path® rings must be seen as another port. Moving it will increase the range of vision. For example pulling it upwards will show the lower part of the rectum (Fig. 7).

### Tip 9

**Fig. 8:**
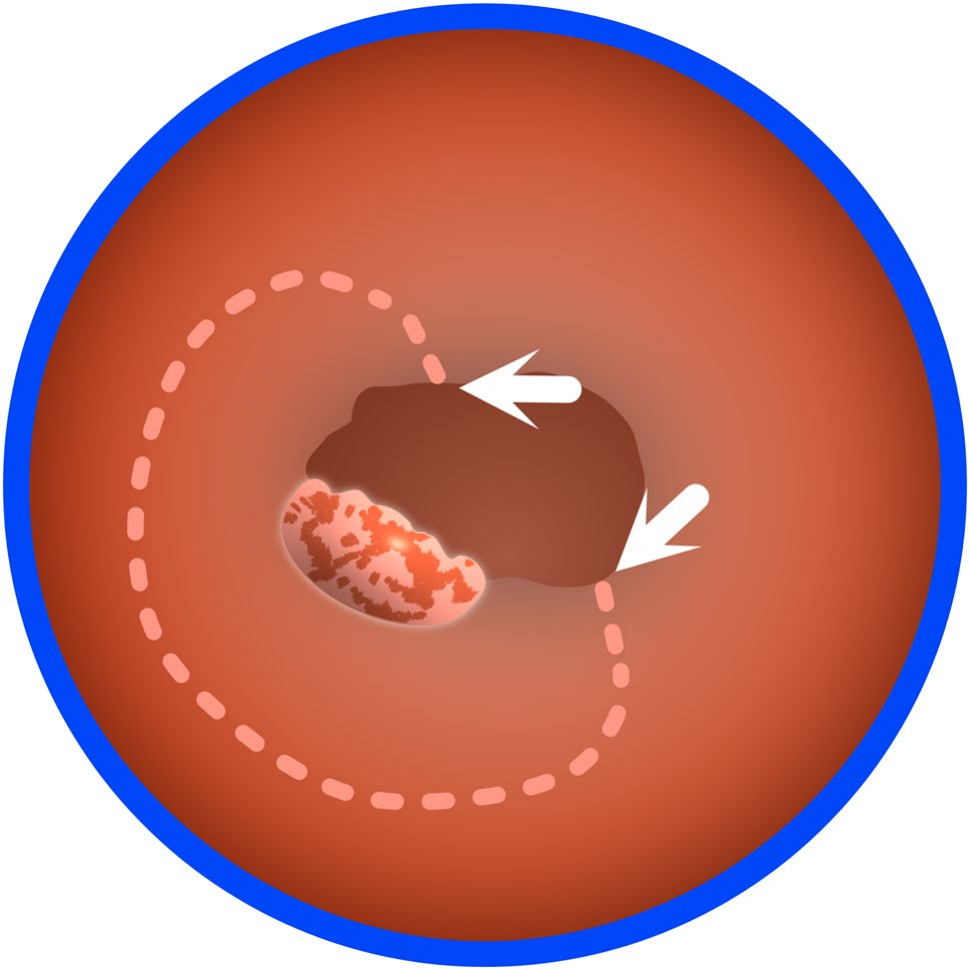
Resection planning

The initial surgical maneuver involves placing marks around the lesion. However, if a stenosis interferes with this process, the first step should be to create space by cutting down the stenosis. This will provide the necessary room to navigate and mark the perimeter of the lesion (Fig. 8).

### Tip 10

Barbed sutures are the preferred choice for closing the defect if needed.

## Instrumentation

We consider the following instruments mandatory for the procedure:

- GelPOINT Path®
- Bipolar forceps
- Monopolar scissors
- Needle holder
- Suction
- Barbed sutures

On the other hand, along our learning curve, we discovered that the following instruments are not absolutely necessary:

- Advanced energy devices: The rectal wall undergoes devascularization because of radiotherapy [15], reducing the risk of hemorrhage during surgery. Consequently, there is no requirement for advanced energy devices.
- Lone Star: Simple fixation to the perianal skin suffices to stabilize the gel port. However, the Lone Star instrument does not improve neumorectum stability, and it may even interfere with the excision of distal tumors. Traction on distal tumors could potentially conceal them beneath the gel port. Ensuring clear visibility during procedures is crucial for accurate diagnosis and treatment.
- Airseal system and GelPoint Path stabilization bag have become redundant with the adoption of the prone position (explained before) and the use of modern insufflators that deliver high airflow. While the Airseal system may retain some utility for smoke extraction, our experience suggests that, in general, it is not required.
- Robotic cutting needle holder. Bipolar forceps may cut threads with energy.

## Conclusions

Adapting existing robotic platforms for narrow anatomical spaces like the anal canal and rectum is challenging. These spaces were not originally designed for such platforms, leading to a high rate of conversions. However, this publication shares valuable insights that have improved our proficiency in mastering this approach.

## Data Availability

No datasets were generated or analysed during the current study.
